# Antidyslipidemic, Antioxidant, and Anti-inflammatory Effects of Jelly Drink Containing Polyphenol-Rich Roselle Calyces Extract and Passion Fruit Juice with Pulp in Adults with Dyslipidemia: A Randomized, Double-Blind, Placebo-Controlled Trial

**DOI:** 10.1155/2022/4631983

**Published:** 2022-09-21

**Authors:** Jurairat Khongrum, Pratoomporn Yingthongchai, Kongsak Boonyapranai, Wachira Wongtanasarasin, Nowwapan Donrung, Wanida Sukketsiri, Aree Prachansuwan, Pennapa Chonpathompikunlert

**Affiliations:** ^1^Science and Technology Research Institute, Chiang Mai University, Chiang Mai 50200, Thailand; ^2^Research Institute for Health Science Chiang Mai University, Chiang Mai 50200, Thailand; ^3^Department of Emergency Medicine, Faculty of Medicine, Chiang Mai University, Chiang Mai 50200, Thailand; ^4^Expert Center of Innovative Health Food and Biodiversity Research Centre, Thailand Institute of Scientific and Technological Research, 12120, Thailand; ^5^Division of Health and Applied Sciences, Faculty of Science, Prince of Songkla University, Songkhla 90110, Thailand; ^6^Institute of Nutrition, Mahidol University, Nakhon Pathom 73170, Thailand

## Abstract

Oxidative stress and inflammation play key roles in the pathophysiology in the pathophysiology of dyslipidemia, which are positive risks that increase atherosclerosis leading to important healthcare problems. Therefore, we aimed to study the antioxidant, anti-inflammatory, and lipid-lowering effects of jelly drink containing polyphenol-rich roselle calyces extract and passion fruit juice with pulp concentrate (RP jelly drink) in comparison to a placebo jelly drink for 8 weeks. Forty-three adults with dyslipidemia were randomly assigned into two groups: the RP jelly drink group and the placebo group. Glucose, total cholesterol (TC) triglyceride (TG), low-density lipoprotein-cholesterol (LDL-C), high-density lipoprotein-cholesterol (HDL-C), oxidative stress biomarkers, inflammatory parameters, and monocyte chemotactic protein-1 (MCP-1) were measured with fasting blood samples at baseline, 4 weeks and 8 weeks of intervention. Results showed a significant decrease in LDL-C and TG, respectively, after 8 weeks of RP jelly drink consumption (LDL-C: 107.63 ± 22.98 *mg*/*dL*; TG: 109.79 ± 38.83 *mg*/*dL*) compared to baseline measurements (LDL-C: 128.43 ± 32.74 *mg*/*dL*; TG: 132.33 ± 75.11 *mg*/*dL*). These may be possible due to reduced inflammation and improvements in oxidative stress, as demonstrated by the reduction of tumor necrosis factor- (TNF-) *α* and malondialdehyde (MDA), and the enhancement of glutathione (GSH) after consuming the RP jelly drink for 8 weeks. However, no significant differences of treatment on glucose, total cholesterol, MCP-1, interleukin-6, and interleukin-10 were observed. In conclusion, daily consumption of RP jelly drink for 8 weeks resulted in significant improvement in lipid profiles in subjects with dyslipidemia. However, more research is needed to assess its nutritional and functional potential.

## 1. Introduction

Cardiovascular disease has become life-threatening worldwide with high morbidity and mortality rates. Positive risk factors can be grouped into metabolic syndromes, including abdominal obesity, high blood pressure, hyperglycemia, and dyslipidemia for the onset of atherosclerosis, which may lead to a series of cardiovascular events [1]. Dyslipidemia is recognized as a risk factor related with atherosclerosis and is characterized by an abnormal lipid profile in which the level of serum cholesterol, triglycerides, or both are elevated, or the level of high-density lipoprotein cholesterol (HDL-C) is reduced [2]. Consumption of foods high in fat and cholesterol and lack of exercise cause high blood lipid levels [3]. Furthermore, the occurrence of increased oxidative stress and inflammation is associated with the regulation of lipid metabolism, and these are major contributors to the incidence of dyslipidemia [4, 5].

The imbalance between oxygen free radicals and antioxidant defenses is negatively altered within cells, on cell membranes and receptors, proteins, lipids, lipoproteins, carbohydrates, and DNA strands [6, 7]. Previous studies have revealed that an increase in lipid peroxidation such as malondialdehyde (MDA) is associated with the serum total cholesterol (TC), triglyceride (TG), and low-density lipoprotein cholesterol (LDL-C) [8]. Likewise, inflammation is recognized as a key role in abnormal lipid metabolism. Higher production of inflammatory cytokines, including tumor necrosis factor- (TNF-) *α* and less potent anti-inflammatory properties such as interleukin-10 (IL-10), induced severe high-density lipoprotein cholesterol (HDL-C) deficiency, LDL-C, and elevated TG. [9, 10]. Furthermore, LDL-C accumulated in the intimal layer of blood vessels was oxidized that caused oxidized low-density lipoproteins (OxLDL) forming in microphages and vascular smooth muscle cells. An excessive OxLDL accumulation could further stimulate proinflammatory signaling pathway because the OxLDL could bind to the monocyte chemoattractant protein-1 (MCP-1) and form a monocyte-attracting lipoprotein which can induce a much stronger chemotactic effect on monocytes than that of OxLDL alone. Either OxLDL or OxLDL-bound MCP-1 play a key role in the initiation and progression of atherosclerosis by promoting the direct migration of inflammatory cell [11]. It is known that lipid-lowering drugs can effectively reduce serum LDL-C levels; however, the adverse effects of the drugs have raised concerns about its use. Therefore, alternative strategies are presented especially in dietary polyphenol and vitamin C that play a role in antioxidant defenses showed high activity of glutathione (GSH) and superoxide dismutase (SOD) along with the reduction of TC, TG, and LDL-C [12–14]. Dietary polyphenols such as fruits, vegetables, legumes, nuts, and plant-derived beverages [15] have received much attention in disease prevention due to their potential therapeutic effects. Roselle calyces (*Hibiscus sabdariffa*) is an important source of vitamins, minerals, and bioactive compounds, such as organic acids, phytosterols, and polyphenols as well [16]. There are many pharmacological actions on the health effects of rosella petals, such as cardioprotective action, antihypertensive effect, and inhibition of LDL-C oxidation [17]. Passion fruit (*Passiflora edulis*) is a tropical fruit composed of polyphenols, flavonoids, alkaloids, carotenoids, tocopherols, and ascorbic acid. These compounds have been recognized for their health effects and biological activity such as antioxidant, antihypertensive, antitumor, antidiabetic, hypolipidemic, and anti-inflammatory activities [14, 18].

However, there are no reports on the benefits of a combination of rosella calyces extract and passion fruit containing bioactive components in functional food products regarding the effects of hypolipidemia. We therefore performed a double-blind randomized, two-arm parallel-group, placebo-controlled trial study to evaluate the hypolipidemic, antioxidant, and anti-inflammatory effects of jelly drink containing polyphenol-rich rosella calyces extract and passion fruit juice with pulp (RP) on repeated intake over a period of 8-week among hyperlipidemic adults.

## 2. Materials and Methods

### 2.1. Study Design

A randomized double-blind, two-arm parallel-group, placebo-controlled trial was conducted with an 8-week intervention among Thai adults with dyslipidemia. The study was performed according to the Good Clinical Practice (GCP) Guideline with fully complied with the ethical guidelines of a clinical trial study according to the Declaration of Helsinki. All procedures involving human subjects were approved by the Health Sciences Human Experimentation Committee under the auspices of the Office of Research Ethics, Research Institute for Health Sciences, Chiang Mai University, Thailand (Code: HSHEC-23/63). All subjects written informed consent before inclusion in the study. The trial was registered at https://www.thaiclinicaltrials.org as TCTR20220326001.

### 2.2. Subjects and Interventions

Forty Thai adults were recruited via poster advertisement from the community around Chiang Mai University. Eligibility criteria were aged 35-60 years, dyslipidemia based on the criteria: serum total cholesterol (*TC* ≥ 200 *mg*/*dL*) and/or triglycerides (*TG* ≥ 150 *mg*/*dL*) and/or LDL cholesterol (*LDL* − *C* ≥ 100 *mg*/*dL*) and/or HDL cholesterol (*HDL*‐*C* < 40 *mg*/*dL*), not currently taking drugs for lowering cholesterol or triglycerides such as statins and fenofibrate and not with a vegan or vegetarian diet. The exclusion criteria included *body* *mass* *index* (*BMI*) > 35 *kg*/*m*^2^, smokers, athletes, diabetes, multiple allergies, traumatic injury, gastrointestinal disease, cancer, central nervous system or psychiatric disorders, and having been treated with herbal medicines and dietary supplements affecting the lipid metabolism in the previous 14 days. The randomization was performed using a computer-generated code with Random Allocation Software. Each subject was blinded and randomly assigned to receive either the RP-jelly drink (300 mL; 100 Kcal) or a placebo jelly drink (300 mL; 100 Kcal) once daily for a period of 8 weeks.

### 2.3. Study Protocol

During the baseline visit, the subject's demographic information and medical history were obtained.

Body weight, height, body mass index (BMI), and blood pressure were assessed individually. Subjects fasted overnight for 10-12 h before venous blood collection. Fasting plasma glucose, serum lipid profile, oxidative stress status, inflammation parameters, and a key chemokine of atherosclerotic lesions were measured before received any treatments, 4-week, and 8-week of intervention. All subjects were asked not to change their dietary and physical activity patterns during the study period.

All subjects were asked not to change their dietary and physical activity patterns during the study period. To ensure the influence of dietary and physical activity patterns on this study, all subjects received a questionnaire about dietary behavior and physical activity patterns. The frequency and quantity of dietary including rice, meat, egg, milk, coconut curry, vegetable, fruits, and dessert at a recalling 7 days before visiting in represent energy intake. The frequency and duration of physical activity including strenuous activities, moderate sport, walking, and sitting at a recalling 7 days before visiting in represent physical activity.

### 2.4. Dietary Interventions

Subjects in the treatment and placebo group were given packs of 300 mL RP jelly drink and placebo jelly drink, respectively. Two products were isocaloric jelly drinks of 100 kcal. The 300 mL RP jelly drink for the treatment group was developed and supported by Expert Center of Innovative Health Food, Thailand Institute of Scientific and Technological Research, Thailand. RP jelly drink was prepared by using water, sweetener, gelling agent, flavoring agent, multivitamin, the roselle extract powder of 0.18%, and the freeze-dried passion fruit juice plus pulp of 0.37%.

Calyces of roselle (*Hibiscus sabdariffa*) were collected from Prachuap Khiri Khan, Thailand, by P. Chonpathompikunlert and authenticated by a taxonomist from the Plant Varieties Protection Office with identification as voucher specimen BK No. 071159 and deposited at the Forest Herbarium, Royal Forest Department, Ministry of Agriculture and Cooperatives, Bangkok, Thailand. Briefly, the roselle calyces were dried in hot air oven 50°C for 24 h. Dehydrated roselle calyces were ground into powder, then mixed with water 1 : 10% (*w*/*v*) ratio, followed by double extraction via maceration method. The aqueous extracts of roselle calyces were filtered (Whatman®, No. 1). The solvent was evaporated to concentrate using stirrer under heat 50°C for 6 h and kept in a freezer, then freeze-dried by the lyophilizer (Alpha 2-4 LSCplus freeze dryer, An der Unteren SÖse, Germany), and the powder was kept in a dark bottle at -20°C until used for RP jelly production. The percentage yield of roselle calyces aqueous extract was 44.53% (*w*/*w*) of the dry powder.

Freeze-dried passion fruit juice plus pulp was obtained from *Passiflora edulis* f. *flavicarpa* Deg. L. plants in Chiang Mai, Thailand, collected by P. Chonpathompikunlert, then authenticated by a taxonomist staff of plant varieties protection office with its identification as A voucher specimen BK No. 082283 and deposited at the Forest Herbarium, Royal Forest Department, Ministry of Agriculture and Cooperatives, Bangkok, Thailand. The purple passion fruit juice and pulp (4.5 kg) was blended, filtered 3 times, then freeze dried, and kept in a dark bottle at -20°C until used for RP jelly production. The percentage yield of the passion fruit juice and pulp was 12.12% (*w*/*w*) of the fresh juice and pulp.

Placebo was prepared by Expert Center of Innovative Health Food, Thailand Institute of Scientific and Technological Research, Thailand, in the same manner of RP jelly without the roselle extract powder and the freeze-dried passion fruit juice plus pulp.

Both RP jelly drink and placebo jelly drink were lyophilized using a Heto Powerdry PL9000 freeze dryer (Allerod, Denmark) at −80°C for 48 h for determination of total phenolic contents using the Folin–Ciocalteu reagent [19], flavonoid content according to spectrophotometric methods based on the formation of aluminium-flavonoid complexes [20], and antioxidant potential using the 2,2′-diphenyl-1-picrylhydrazyl (DPPH) radical scavenging method [21].

Data of total phenolic contents, flavonoid content, and antioxidant activity were presented as the mean of five separate experiments and error bars are displayed with standard error. In order to compare the difference in total phenolic contents, flavonoid contents, and antioxidant activity between RP jelly drink and placebo jelly drink, the statistical significance of the data was analyzed using an independent *t*-test. *P* value < 0.05 was considered as statistical significance, and the results are shown in Table 1.

In addition, they were identified and quantified the contents of gallic acid, quercetin, and ascorbic acid using high-performance liquid chromatography (HPLC) with Agilent Por shell 120 EC-C18, 4.6 × 100 *mm*, 2.7 *μ*m column (Agilent 1260 Infinity LC system, Waldbronn, Germany). The separation was performed using ternary linear elution gradient with 20 mM KH2PO4, 60% methanol, and 40% acetonitrile at 284 nm. Standards were run in similar conditions of chromatography to match the retention items [22] and to calculate the quantification.

### 2.5. Blood Analysis

Whole blood samples were collected intravenously by a registered nurse into sodium fluoride containing tubes and clotted blood tubes. The separated plasma/serum determined the biochemical results including glucose, TG, TC, HDL-C, and LDL-C at Chiang Mai Medical Lab, Chiang Mai, Thailand. Another sample of whole blood was collected into an EDTA plasma tube for oxidative stress, antioxidant activity, inflammatory markers, and MCP-1, placed immediately on ice, and centrifuged within 30 min (3600 rpm for 10 minutes at 4°C) to separate plasma. Plasma then was stored at -80°C until analysis.

Oxidative stress was evaluated by lipid peroxidation level as thiobarbituric acid reactive substances (TBARS) method. Malondialdehyde (MDA) assay was adapted from the procedure previously described [23]. The plasma was prepared by mixing with 20% trichloroacetic acid (TCA) and incubated for 15 min at room temperature. The supernatant of mixed plasma was collected after centrifugation. Thiobarbituric acid (TBA) was added to supernatant sample in test tube before boiling at 100°C for 30 min for measuring lipid peroxidation. The MDA value was determined at wavelength of 530 nm and expressed in nmol/L.

Antioxidant activity was determined by glutathione (GSH) level according to the procedure previously described [24]. The assay of GSH with DTNB was performed by following the standard method of Ellman [25] and slightly modified method of Tipple and Rogers [24]. A 20 *μ*L of sample was added into 96-well plate with 10 *μ*L of solution of 10 mM NaH2PO4 and 1 mM dithiothreitol, 100 *μ*L of 1 mM sodium azide dissolved 40 mM potassium phosphate buffer (pH 7.0), 10 *μ*L of 50 mM glutathione, and 100 *μ*L of 30% H2O2. Then, it was shaken for 10 minutes before adding 10 *μ*L of 10 mM 5,5-dithiobis-2-nitrobenzoic acid (DTNB) and immediately measured for absorbance. Later, the GSH level in samples was calculated using A412 nm from standard curve of GSH concentration.

Inflammatory markers including IL-10, IL-6 and TNF-alpha were measured by enzyme-linked immunosorbent assay kit using paired antibodies. (Abcam®, Waltham, MA, USA, for IL-10 and Elabscience®, Houston, Texas, USA, for IL-6 and TNF-*α*).

A key chemokine of atherosclerotic lesion, MCP-1 expression, was also evaluated via enzyme linked immunosorbent assay kit (Elabscience®, Houston, Texas, USA).

### 2.6. Statistical Analysis

#### 2.6.1. Sample Size

A total sample size of 40 subjects was estimated based on calculation of sample size by comparing two means according to sample size estimation in clinical trial [26] with a power of 80%, a significant level of 0.05, and dropout of 10%.

#### 2.6.2. Data Analysis

A statistician was blinded to analyze the results of randomized participants who have received RP jelly drink and placebo treatment. Statistical analyses were being conducted by SPSS software version 22 (SPSS Inc., Chicago, IL, USA) for windows licensed, Chiang Mai University. The minimum level of statistical significance was set to *P* < 0.05.

The data of total phenolic compounds, flavonoid, and antioxidant activity were expressed as *means* ± *standard* *deviation* of at least three replicates. The difference when compared between RP jelly drink and placebo product was analyzed by independent *t*-test.

For continuous variables, we expressed data as *mean* ± *standard* *deviation* and coefficient of variation. Kolmogorov-Smirnov test was applied to ensure the normal distribution of variables, and nonnormally distributed data were log-transformed. Differences between the intervention and placebo groups were analyzed on an intention-to-treat (ITT) basis. Missing data was imputed following the last observation carried forward (LOCF) principle. We performed sensitivity analysis with per protocol (PP) principle in which the missing values were not imputed (*n* = 20 in placebo group, *n* = 20 in RP jelly drink group). The comparability of general characteristics of subjects at baseline was assessed by independent *t*-test. A repeated measure one-way ANOVA with a Greenhouse-Geisser correction and followed by the Bonferroni *post hoc* test in pairwise comparisons was performed to identify significant differences at different consumption (treatment) times and to analyze main effective differences between groups.

For quantitative data analysis, dietary intake and physical activity questionnaire were used for monitoring the subject's nutrition intake and energy expenditure. We expressed data as *mean* ± *standard* *deviation* in the range of a data set, and independent *t*-test was performed to analyze the significant difference of between groups.

## 3. Results

### 3.1. Bioactive Compounds and Antioxidant Activities of RP Jelly Drink

**Figure 1:**
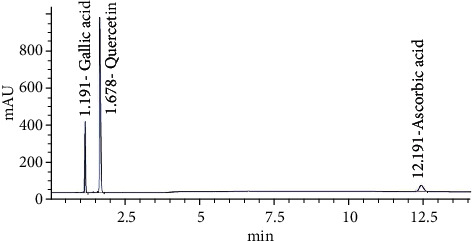
HPLC chromatogram of RP jelly drink for gallic acid, quercetin, and ascorbic acid.

The chromatographic profiles of the RP jelly drink showed three types of the phytoconstituents, gallic acid, quercetin, and ascorbic acid (Figure 1). The retention time of gallic acid, quercetin, and ascorbic acid in RP jelly drink was found to be 1.191, 1.678, and 12.191, and it matched with standard retention time values, respectively. The amount of gallic acid, quercetin, and ascorbic acid in RP jelly drink was found to be 0.001 ± 0.000 *μg*/*mg*, 0.02 ± 0.000 *μg*/*mg*, and 0.0006 ± 0.000 *μg*/*mg*. Total phenolic compounds, flavonoid contents, and antioxidant activity of the RP jelly drink are shown in Table 1. The data showed that total phenolic contents and flavonoid contents and antioxidant activity by DPPH radical scavenging of RP jelly drink were 1.97 ± 0.03 *mg* GAE/g DW and 1.79 ± 0.20 *mg* QE/g DW, respectively. Antioxidant activity by DPPH radical scavenging shown in IC_50_ value was 2.87 ± 0.05 *mg*/*mL* jelly drink.

### 3.2. General Characteristics of Subjects

**Figure 2:**
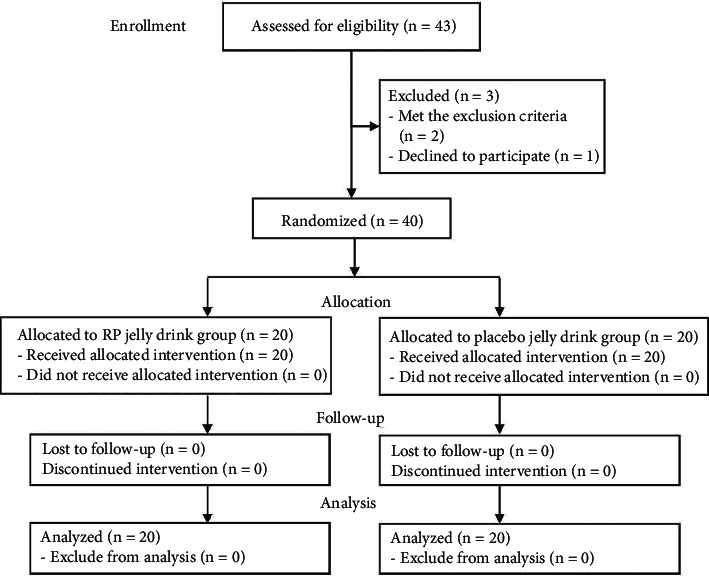
CONSORT flow diagram for the study.

A total of 43 subjects were enrolled in the study as shown in Figure 2. Two subjects met the exclusion criteria, and one subject declined to participate the study. Finally, 40 subjects (20 RP jelly drink; 20 placebo jelly drink) were included, and no withdrawal events were observed. Subjects were 15 women and 5 men in RP jelly group and 14 women and 6 men in placebo jelly drink group. General characteristics of subjects participated in the study are summarized in Table 2.

### 3.3. Effect of Jelly Drinks on Lipid Profiles, Inflammatory Markers, and Other Parameters

Effects of jelly drinks on lipid profiles, inflammatory markers, and other parameters within each group at different times are shown in Table 3. After a 4-week consumption period, the subjects who consumed RP jelly drinks showed a significant reduction in LDL-C (*P* < 0.05) and an enhancement of HDL-C (*P* < 0.05). Noticeably, LDL-C and TG were significantly reduced after 8 weeks of RP jelly drink consumption (16% and 17%, respectively), while LDL-C was unchanged, and it was likely that TG increased over the time of the placebo group. However, after 4 and 8 weeks of intervention, no differences were found in BMI, blood pressure, fasting plasma glucose, and TC in each study group (Table 3).

After consuming the RP jelly drink, plasma MDA was significantly lower (*P* < 0.001) at the time of the intervention and noticed a significant difference compared to the placebo group. With respect to antioxidant enzymes, subjects who drank the RP jelly products showed significantly higher plasma GSH levels at the 4 weeks and 8 weeks of the study period compared to baseline measurements. This change was seen only in the RP jelly drink group than in the placebo group.

Inflammatory cytokines, including IL-6, TNF-*α*, and IL-10, which may contribute to the acceleration of abnormal lipid metabolism, have also been evaluated. Results revealed that those who consumed the RP jelly drink had a significant reduction in TNF-*α* levels at 8 weeks of the study duration (*P* < 0.05). No significant changes in IL-6, IL-10, and MCP-1 levels were observed after consuming the RP jelly drink.

Energy intake and physical activity did not change over time or between groups during the study period (Tables 4 and 5).

## 4. Discussion

In this randomized, double-blind controlled trial, consuming a jelly drink containing polyphenol-rich roselle extract and passion fruit juice plus pulp concentrate (RP jelly drink) once daily for 8 weeks, clearly demonstrated a significant reduction in LDL-C and TG levels when compared to either baseline measurements or jelly drink placebo in Thai adults with dyslipidemia, whereas the changes in fasting plasma glucose, total cholesterol, HDL-C, MCP-1, interleukin-6, and interleukin-10 indicated no significant difference between the RP jelly drink and the placebo group. Furthermore, we also found lower inflammatory markers and improved oxidative stress, which were seen in the reduction of tumor necrosis factor- (TNF-) *α* and malondialdehyde (MDA), and the enhancement of glutathione (GSH) after the ingestion of the RP jelly drink for 8 weeks. This investigation emphasized the impact of a polyphenol-rich product supplementation on the beneficial effect of lowering blood lipids resulting in a protective effect against atherosclerosis that might help to primary prevention of cardiovascular disease.

Numerous observational studies indicated that polyphenol-rich foods were strongly associated with a lower incidence of cardiovascular diseases, metabolic syndrome, and diabetes [27–29]. Consistency with knowledge that Passion fruit and roselle calyces have been well-documented to improve serum HDL-C and reduce LDL-C levels [30–32]. This might be associated with the presence of quercetin and gallic acid [33–35] in RP jelly drink consumption. Additionally, a recent meta-analysis study of 34 randomized controlled trials reported that consuming polyphenol-containing supplements significantly improved blood lipid levels (LDL-C: -4.39 mg/dL, *P* = 0.009; HDL-C: 2.68 mg/dL, *P* < 0.001) in comparison with the placebo group [36]. This observation may be related to lipoprotein metabolism by increasing fecal cholesterol elimination and impeding apolipoprotein production [36] or enhancement of the cholesterol efflux capacity [37]. Another possible mechanism may be involved with the effect of intestinal microbiota on lipid homeostasis by increasing polyphenol bioavailability and promoting polyphenol metabolites production, which includes bile acids, lactic acids, and short-chain fatty acids [38, 39]. These studies support that polyphenol-containing products may benefit people with high cholesterol levels or those at risk of heart diseases.

It has been suggested that the polyphenols, anthocyanins, and other bioactive compounds found in roselle calyces (*Hibiscus sabdariffa*) may contribute to lipid-lowering and inhibition of LDL-C oxidation through its antioxidant activity or other mechanisms [40]. A human trial conducted by Lin et al. [41] revealed that taking one or two capsules of roselle extract reduced TC by 11-15% after 4 weeks of supplementation; however, there were no reports for LDL-C, HDL-C, and TG. In a recent meta-analysis of 9 clinical trials, the efficacy of roselle supplementation in regulating cholesterol levels in patients with metabolic syndrome and related diseases showed a reduction in total cholesterol, LDL-C, and HDL-C but not TG [42]. In our study, daily consumption of an RP jelly drink containing polyphenol-rich roselle extract for 8 weeks tends to have a positive effect on the changes in LDL-C, TG, TC, and HDL-C, levels as time passed; nevertheless, no significant differences were found in TC and HDL-C between study groups. Our results were consistent with a meta-analysis studied by Aziz et al. [43], when we focused on the pooled results of two placebo-controlled studies [44, 45]. Nonetheless, this meta-analysis concluded that the limited evidence from clinical studies cannot support the effect of *Hibiscus sabdariffa* on blood lipids.

For passion fruit, an animal study performed by Souza et al. 2012 [30] revealed that the treatment group that received concentrated passion fruit juice twice daily for 28 days had better antioxidant activity and lipid profiles compared to the control group. Similar to another study, passion fruit juice was be able to improve lipid and blood glucose levels in offspring from diabetic and nondiabetic mothers of Wistar rats [46]. These effects may be attributed to the presence of flavonoids, consequently showing antioxidant and anti-inflammatory properties derived from passion fruit juice. Interestingly, several *in vivo* studies have shown that flavonoids have a strong inhibitory effect on 3-hydroxy 3-methylglutaryl coenzyme A (HMG-CoA) reductase and increased activity of lecithin cholesterol acyltransferase (LCAT); thereby, changes in blood lipid levels were observed [47, 48]. It might be one of the explanations why RP jelly drink, which combined passion fruit juice and roselle calyces, demonstrated a significant reduction in LDL-C and TG levels. However, the efficacy of *Passiflora edulis* juice alone has not been investigated in human clinical studies.

Oxidative stress has been associated with many pathologies especially cardiovascular disease. The balance between reactive oxygen species (ROS) and antioxidants are important to prevent the pathological progression [49]. With regard to the reduction of malondialdehyde (MDA) and the enhancement of glutathione (GSH), the present findings supported that consuming RP jelly drinks can improve oxidative stress in the body. It is well-known that polyphenols play an important role in inhibiting oxidative stress and are also connected to anti-inflammatory effects in order to remain cellular homeostasis [50]. Oxidative stress and inflammation are not only related to dyslipidemia but also contribute to the onset and progression of atherosclerosis [51]. As we have known that TNF-*α* can trigger the cytokine cascade and contribute to regulate the production of another inflammatory cytokine [52, 53]. Remarkably, the abnormality of TNF-*α* level and signaling cause the development of disease, such as rheumatoid arthritis, psoriasis, Crohn's disease, atherosclerosis, and cancer [53]. In our study, plasma tumor necrosis factor- (TNF-) *α* was decreased after the ingestion of the RP jelly drink for 8 weeks. This observation may be explained by the natural active ingredients presented in RP jelly drinks exert anti-inflammatory effects through multitargeted action in the inflammatory pathway [54]. Consistent with the study of Herranz-Lopez et al. (2017), the effects of *Hibiscus sabdariffa*-derived polyphenols on specific cellular pathways have been reviewed, particularly in pathways associated with chronic inflammation and energy metabolism [55]. A metabolomics and gene-expression study has demonstrated that *Hibiscus sabdariffa* polyphenols can downregulate genes involved in cholesterol and TG synthesis and exert a multitarget lipid-lowering effect [56]. In addition, it has been proposed that TNF-*α* itself can suppress free fatty acid uptake, inhibit the activity of enzymes involved in lipid metabolism, and regulate cholesterol metabolism [57]. Moreover, Passion fruit has been evaluated to have anti-inflammatory activity by reducing the level of proinflammatory IL-1*β* and TNF-*α* in an *in vivo* study [58, 59] and decrease IL-17A in human [32]. This could be attributed to the presence of bioactive compounds like C-glycosyl flavonoids vicenin, orientin, isoorientin, vitexin, and isovitexin [59]. Therefore, the reductions in TNF-*α*, LDL-C, and TG as seen in our study appeared to be consistent.

This study has some strengths. First, the double-blind, placebo-controlled study was conducted with the subjects with no differences in baseline general characteristics between groups. Participants in this study did not require a wash-out period between placebo and treatment as a cross-over design, resulting in no dropping out from the trial. Second, two kinds of jelly drinks (RP jelly drink and placebo) were prepared with isocaloric and iso-macronutrients (carbohydrates, proteins, and fats) in the same format. Roselle extract powder and the dried passion fruit juice with pulp are used only in RP jelly drink which is different from placebo; hence, we can ensure the effectiveness of the intervention provided in the lipid-lowering function. Nevertheless, there are limitations. First of all, due to the study not being conducted in an inpatient setting, the participants' diet and physical activity could not be rigorously controlled during the study period. Natural polyphenols or other bioactive substances presented in common diets may interfere with our study. However, we asked participants to refrain from changing their diet and exercise patterns throughout the 8-week study. Secondly, the long-term effects of RP jelly drink supplementation on lipid-lowering and other relevant clinical endpoints should be performed. Thirdly, several factors including short duration of the study, a lack of consideration of sex-related effects, homogenous ethnic background, and the relatively small study numbers might associate with the lipid-lowering effect. Therefore, any direct cause-effect relationships or precise mechanisms including reduced lipid content of 3-hydroxyl-3-methylglutaryl coenzyme A reductase (HMGCR) activity and sterol regulatory element binding protein-2 (SREBP2) also require further investigation.

## 5. Conclusion

**Figure 3:**
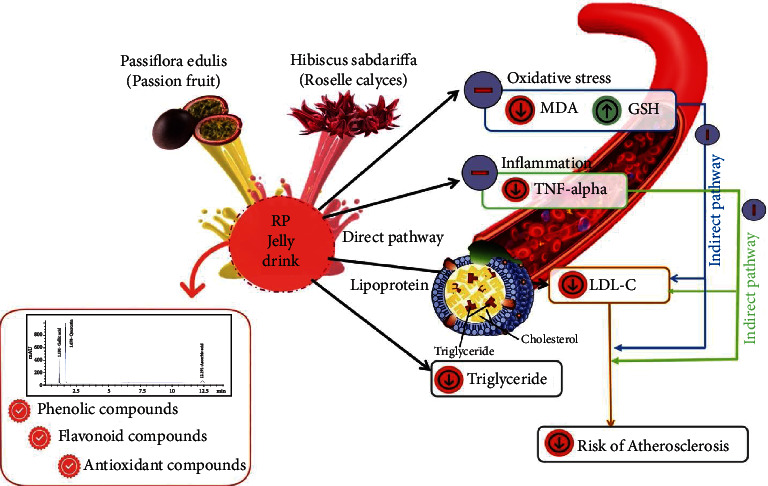
The framework of potential components in RP jelly drink.

Our findings indicated that once-daily consumption of RP jelly drink for eight weeks improved blood lipid profiles compared to placebo among Thai adults with dyslipidemia in uncontrolled nutrition intake. The possible underlying mechanism of improved blood lipid levels is unclear. However, it can be concluded that a RP jelly drink product, which contains large amounts of phytoconstituents (quercetin, gallic, and ascorbic acid), total phenolic, and flavonoid compound exhibiting antioxidant activities could represent another polyphenol-rich food product with antioxidant and anti-inflammatory effects, which in turn may indirectly enhance lowering blood lipid levels resulting in decreased risk of the initiation of atherosclerosis (Figure 3). Further study of the nutritional qualities of RP jelly drink and the mechanisms underlying its action in reducing lipids in the body should be performed to evaluate its potential role in the prevention of cardiovascular disease.

## Figures and Tables

**Table 1:** The total phenolic contents, flavonoids contents, and antioxidant activity of RP jelly drink and placebo jelly drink.

Parameters	RP jelly drink	Placebo jelly drink	*P* value
Total phenolic content, mg GAE/g DW	1.97 ± 0.03	0.98 ± 0.01	<0.001
Flavonoid content, mg QE/g DW	1.79 ± 0.20	0.87 ± 0.12	0.002
DPPH (*IC*_50_), mg/mL	2.87 ± 0.05	4.79 ± 0.02	<0.001

Data are expressed as *mean* ± *standard* *deviations*. Data were analyzed using independent *t-*test for comparing the parameters between study groups. GAE: gallic acid equivalent; DW: dried weight; QE: quercetin equivalent; DPPH: 2, 2-diphenyl-1-picrylhydrazyl.

**Table 2:** General characteristics of subjects participated in the study (*n* = 40).

Parameters	RP jelly drink (*n* = 20)	Placebo jelly drink (*n* = 20)	*P* value
AGE, years	37.00 ± 3.61	36 ± 6.14	0.469
BMI, kg/*m*^2^	24.685 ± 4.35	24.145 ± 4.53	0.881
SBP, mmHg	119.58 ± 11.82	118.90 ± 3.47	0.875
DBP, mmHg	71.48 ± 2.01	69.43 ± 1.92	0.712
FPG, mg/dL	79.50 ± 13.76	78.28 ± 2.71	0.702
TC, mg/dL	203.76 ± 38.04	199.8 ± 35.12	0.731
TG, mg/dL	132.33 ± 75.11	130.73 ± 29.50	0.425
HDL-C, mg/dL	47.00 ± 14.40	49.70 ± 10.62	0.500
LDL-C, mg/dL	128.43 ± 32.74	126.75 ± 31.76	0.869

Data are expressed as mean ± standard deviations. Data were analyzed using independent *t-*test for comparing the parameters between study groups. BMI: body mass index; SBP: systolic blood pressure; DBP: diastolic blood pressure; FPG: fasting plasma glucose; TC: total cholesterol; TG: triglyceride; HDL-C: high-density lipoprotein cholesterol; LDL-C: low-density lipoprotein cholesterol.

**Table 3:** Lipid profiles, inflammatory markers, and other parameters of the subjects within each group at different times and between groups.

Parameters	RP jelly drink (*n* = 20)			*P* value within group	Placebo jelly drink (*n* = 20)			*P* value within group	*P* value between group
	Baseline (CV%)	4 weeks (CV%)	8 weeks (CV%)		Baseline (CV%)	4 weeks (CV%)	8 weeks (CV%)		
BMI, kg/m^2^	24.685 ± 4.35 (17.62)	24.42 ± 4.84 (19.82)	24.34 ± 3.82 (15.69)	0.895	24.145 ± 4.53 (18.02)	24.9 ± 4.27 (17.15)	24.81 ± 4.34 (17.49)	0.832	0.950
SBP, mmHg	119.58 ± 11.82 (9.89)	116.25 ± 17.57 (15.11)	112.21 ± 31.09 (27.97)	0.543	118.90 ± 3.47 (2.92)	110.00 ± 28.38 (21.25)	115.95 ± 16.55 (14.27)	0.230	0.945
DBP, mmHg	71.48 ± 2.01 (2.81)	78.55 ± 15.34 (19.53)	79.89 ± 15.89 (19.89)	0.818	69.43 ± 1.92 (2.77)	76.35 ± 8.40 (11.00)	77.84 ± 12.29 (15.79)	0.234	0.464
FPG, mg/dL	79.50 ± 13.76 (17.35)	82.25 ± 18.52 (22.52)	81.58 ± 15.12 (14.11)	0.094	78.28 ± 2.71 (3.46)	79.15 ± 11.91 (15.05)	80.68 ± 17.08 (21.17)	0.506	0.357
TC, mg/dL	203.76 ± 38.04 (18.67)	198.35 ± 35.59 (17.94)	192.79 ± 27.08 (14.05)	0.727	199.8 ± 35.12 (17.57)	214.62 ± 39.12 (18.23)	201.95 ± 43.54 (21.56)	0.214	0.530
TG, mg/dL	132.33 ± 75.11 (56.76)	142.55 ± 54.00 (37.88)	109.79 ± 38.83^a^ (36.28)	0.020^∗^	130.73 ± 29.50 (22.57)	164.95 ± 41.85^a^ (25.37)	145.84 ± 48.90 (33.53)	0.775	0.016^∗^
HDL-C, mg/dL	47.00 ± 14.40 (30.64)	55.81 ± 10.35^a^ (18.55)	51.29 ± 10.52 (20.51)	0.014^∗^	49.70 ± 10.62 (21.37)	51.70 ± 11.45 (22.15)	51.11 ± 11.24 (21.99)	0.447	0.780
LDL-C, mg/dL	128.43 ± 32.74 (25.49)	107.47 ± 20.87^aa^ (19.42)	107.63 ± 22.98^aa^ (21.35)	0.0.29^∗^	126.75 ± 31.76 (25.06)	133.39 ± 31.49 (23.61)	128.11 ± 34.56 (26.98)	0.766	0.018^∗^
MDA, nmol/mL	0.516 ± 0.032 (6.20)	0.415 ± 0.010^a^ (2.41)	0.471 ± 0.024 (5.10)	0.001^∗∗∗^	0.524 ± 0.042 (8.02)	0.512 ± 0.016 (3.13)	0.553 ± 0.021 (3.80)	0.493	0.032
GSH, *μ*g/mL	0.554 ± 0.084 (15.16)	1.024 ± 0.141^a^ (13.77)	1.09 ± 0.148^a^ (14.52)	0.091	0.577 ± 0.072 (12.48)	0.607 ± 0.048 (7.91)	0.428 ± 0.053 (12.38)	0.518	0.020^∗^
TNF-*α*, pg/mL	14.75 ± 1.97 (13.36)	12.47 ± 1.89 (15.16)	6.06 ± 0.34^a^ (5.61)	0.025^∗^	13.68 ± 2.18 (15.94)	14.05 ± 1.92 (13.67)	11.19 ± 1.15 (10.28)	0.586	0.026
IL-6, pg/mL	1.02 ± 0.10 (9.80)	1.04 ± 0.05 (18.29)	1.05 ± 0.09 (8.57)	0.810	0.95 ± 0.06 (6.32)	1.04 ± 0.13 (12.50)	1.05 ± 0.06^a^ (15.70)	0.003^∗∗^	0.096
IL-10, pg/mL	5.62 ± 0.96 (17.08)	6.07 ± 1.11 (13.77)	7.07 ± 1.04 (14.71)	0.516	5.34 ± 0.73 (13.67)	6.06 ± 1.61 (26.57)	7.09 ± 0.95 (13.40)	0.638	0.854
MCPL-1, pg/mL	7.77 ± 0.70 (9.91)	8.51 ± 0.38 (4.46)	7.14 ± 0.72 (10.08)	0.193	7.38 ± 0.34 (4.61)	9.43 ± 0.28 (2.97)	7.65 ± 0.72 (9.41)	0.585	0.673

Data expressed as *mean* ± *standard* *deviations* and the coefficient of variation percent (CV%). Data were analyzed using repeated-measured ANOVA for comparing the changes over time within and between study groups. ^a^ indicates the significant differences within each group at different times when compared to baseline (*P* value <0.05). BMI: body mass index; SBP: systolic blood pressure; DBP: diastolic blood pressure; FPG: fasting plasma glucose; TC: total cholesterol; TG: triglyceride; HDL-C: high-density lipoprotein cholesterol; LDL-C: low-density lipoprotein cholesterol; MDA: malondialdehyde; GSH: glutathione reduced; IL: interleukin; TNF-*α*: tumor necrosis factor alpha; MCP-1: monocyte chemoattractant protein-1.

**Table 4:** Frequency and quantity of dietary items including rice, meat, eggs, milk, coconut-based curry, vegetable, fruits, and dessert at as recalled for 7 days prior to visit (baseline, 4 weeks, and 8 weeks).

Dietary		Treatment group	Baseline	*P* value	4 weeks	*P* value	8 weeks	*P* value
Rice	Frequency (score)	Placebo	5.65 ± 0.11	0.230	5.76 ± 0.10	0.617	5.76 ± 0.11	0.284
		RP jelly drink	5.48 ± 0.10		5.84 ± 0.15		5.44 ± 0.20	
	Quantity (score)	Placebo	2.64 ± 0.14	0.302	2.63 ± 0.13	0.248	2.65 ± 0.13	0.610
		RP jelly drink	2.40 ± 0.13		2.36 ± 0.14		2.36 ± 0.13	
Meat	Frequency (score)	Placebo	4.76 ± 0.23	0.559	4.76 ± 0.27	0.403	5.18 ± 0.23	0.930
		RP jelly drink	4.84 ± 0.25		4.92 ± 0.32		5.20 ± 0.19	
	Quantity (score)	Placebo	1.76 ± 0.14	0.922	1.64 ± 0.13	0.852	1.80 ± 0.12	0.707
		RP jelly drink	1.80 ± 0.13		1.76 ± 0.14		1.68 ± 0.13	
Egg	Frequency (score)	Placebo	3.36 ± 0.32	0.683	2.96 ± 0.35	0.527	2.92 ± 0.31	0.579
		RP jelly drink	3.20 ± 0.34		2.86 ± 0.35		2.88 ± 0.21	
	Quantity (score)	Placebo	2.36 ± 0.14	0.566	2.24 ± 0.19	0.693	2.16 ± 0.16	0.438
		RP jelly drink	2.60 ± 0.12		2.44 ± 0.14		2.48 ± 0.13	
Milk	Frequency (score)	Placebo	3.84 ± 0.34	0.907	3.52 ± 0.33	0.936	3.28 ± 0.33	0.898
		RP jelly drink	3.72 ± 0.36		3.22 ± 0.40		3.10 ± 0.24	
	Quantity (score)	Placebo	2.20 ± 0.13	0.895	1.92 ± 0.16	0.687	1.88 ± 0.12	0.895
		RP jelly drink	2.32 ± 0.16		1.92 ± 0.17		2.16 ± 0.11	
Coconut curry	Frequency (score)	Placebo	1.96 ± 0.38	0.149	1.94 ± 0.43	0.836	1.92 ± 0.36	0.535
		RP jelly drink	1.84 ± 0.36		1.88 ± 0.40		1.88 ± 0.38	
	Quantity (score)	Placebo	1.22 ± 0.20	0.139	1.16 ± 0.21	0.994	1.00 ± 0.17	0.885
		RP jelly drink	1.64 ± 0.14		1.16 ± 0.21		1.04 ± 0.18	
Vegetable	Frequency (score)	Placebo	4.44 ± 0.36	0.741	5.08 ± 0.29	0.148	4.88 ± 0.32	0.615
		RP jelly drink	5.04 ± 0.20		5.24 ± 0.19		4.84 ± 0.19	
	Quantity (score)	Placebo	1.84 ± 0.14	0.230	1.88 ± 0.13	0.627	1.80 ± 0.13	0.193
		RP jelly drink	2.24 ± 0.16		1.96 ± 0.15		1.88 ± 0.12	
Fruits	Frequency (score)	Placebo	3.46 ± 0.37	0.695	3.84 ± 0.30	0.920	3.84 ± 0.25	0.935
		RP jelly drink	3.42 ± 0.34		3.80 ± 0.33		3.88 ± 0.25	
	Quantity (score)	Placebo	1.78 ± 0.18	0.870	1.80 ± 0.10	0.334	1.80 ± 0.13	0.783
		RP jelly drink	1.76 ± 0.21		2.08 ± 0.15		1.84 ± 0.11	
Dessert	Frequency (score)	Placebo	1.38 ± 0.35		1.08 ± 0.37	0.696	1.84 ± 0.35	0.911
		RP jelly drink	1.76 ± 0.39	0.279	1.00 ± 0.41		1.04 ± 0.36	
	Quantity (score)	Placebo	1.36 ± 0.15		1.56 ± 0.18	0.450	1.48 ± 0.14	0.580
		RP jelly drink	1.24 ± 0.14	0.219	1.48 ± 0.15		1.40 ± 0.15	

*P* value is a statistical measurement value used to represent the difference between groups by independent *t*-test. Data were expressed as *mean* ± *SD* in the range of a data set. Frequency scores were calculated from a ranking of 0 = not consume, 1 = 1-2 times a week, 2 = 3-4 times a week, 3 = 5-6 times a week, 4 = 1 time a day, 5 = 2 times a day, 6 = 3 times a day, and 7 = more than 3 times a day. Quantity score was calculated from a ranking of 1 = less than a half rice plate, 2 = a half plate to 1 plate, 3 = 1 and a half plate, and 4 = more than 1 and a half plate.

**Table 5:** The frequency and duration of physical activity including strenuous activities, moderate sport, walking, and sitting as recalled for 7 days prior to visit (baseline, 4 weeks, and 8 weeks).

Activities		Treatment group	Baseline	*P* value	4 weeks	*P* value	8 weeks	*P* value
Strenuous activities	Frequency	Placebo	3.40 ± 0.30	0.707	3.16 ± 0.36	0.943	3.44 ± 0.35	0.974
		RP jelly drink	3.35 ± 0.36		3.28 ± 0.33		3.28 ± 0.33	
	Duration	Placebo	1.64 ± 0.43	0.880	2.48 ± 0.35	0.799	2.28 ± 0.34	0.981
		RP jelly drink	1.83 ± 0.27		2.68 ± 0.42		2.24 ± 0.36	
Moderate sport	Frequency	Placebo	1.64 ± 0.36	0.957	3.56 ± 0.32	0.088	3.60 ± 0.34	0.432
		RP jelly drink	4.00 ± 0.31		3.80 ± 0.34		3.32 ± 0.33	
	Duration	Placebo	1.40 ± 0.28	0.534	1.92 ± 0.23	0.265	1.64 ± 0.18	0975
		RP jelly drink	1.58 ± 0.18		1.76 ± 0.20		1.68 ± 0.19	
Walking	Frequency	Placebo	6.88 ± 0.12	0.738	6.92 ± 0.08	0.957	6.88 ± 0.12	0.794
		RP jelly drink	6.56 ± 0.23		6.96 ± 0.04		6.92 ± 0.08	
	Duration	Placebo	2.20 ± 0.39	0.391	2.28 ± 0.34	0.562	1.76 ± 0.19	0.445
		RP jelly drink	2.33 ± 0.39		1.68 ± 0.20		1.88 ± 0.22	
Sitting	Frequency	Placebo	6.52 ± 0.16	0.568	6.80 ± 0.14	0.316	6.88 ± 0.12	0.770
		RP jelly drink	6.56 ± 0.22		6.96 ± 0.04		6.92 ± 0.08	
	Duration	Placebo	4.36 ± 0.46	0.468	3.60 ± 0.39	0.676	3.12 ± 0.28	0.585
		RP jelly drink	3.25 ± 0.39		3.00 ± 0.37		2.76 ± 0.27	

*P* value is a statistical measurement value used to represent the difference between groups by independent *t-*test. Data were expressed as mean ± SD in the range of a data set. Frequency scores were calculated from a ranking of 0 = not activity, 1 = 1-2 times a week, 2 = 3-4 times a week, 3 = 5-6 times a week, 4 = 1 time a day, 5 = 2 times a day, 6 = 3 times a day, and 7 = more than 3 times a day. Quantity score of quantity was calculated from ranking of 0 = not activity, 1 = 1-10 minutes, 2 = 10-20 minutes, 3 = 20-30 minutes, 4 = 30-40 minutes, 5 = 40-50 minutes, 6 = 50-60 minutes, and 7 = more than 1 hour.

## Data Availability

All data supporting the conclusions of this work were included within the article. Further inquiries can be directed to the corresponding authors.
